# A Tillaux Fracture and Concurrent Nondisplaced Salter-Harris Type III Fracture of the Distal Fibula: A Case Report

**DOI:** 10.7759/cureus.39651

**Published:** 2023-05-29

**Authors:** Mario Giacobazzi, Makayla Gologram, Robert Mitchell, Connor Kasik, Noah M Gonzalez

**Affiliations:** 1 Orthopedic Surgery, Lake Erie College of Osteopathic Medicine, Erie, USA; 2 Orthopedic Surgery, Illinois Valley Orthopedics, St. Margaret’s Health, Peru, USA

**Keywords:** orthopedic surgery, pediatric fractures, pediatric orthopedic surgery, distal fibula fracture, salter harris type 3, tillaux fracture

## Abstract

The Salter-Harris classification system categorizes pediatric fractures in relation to the physis. A Salter-Harris type III fracture occurs from the physis extending to the epiphysis. Tillaux fractures are a type of Salter-Harris type III fracture that occurs due to incomplete fusion of the growth plate and includes the anterolateral tibial epiphysis. This specific fracture is unique to adolescents due to the anterior tibiofibular ligament's strength in relation to the growth plate, causing avulsion of the tibial fragment. The settings for a Tillaux fracture and a Salter-Harris type III fracture are uncommon due to the mechanism of injury, and it is incredibly rare to have two separate fractures of these classifications in the same ankle. In this case study, a 16-year-old male presented to the emergency department after sustaining trauma to the right ankle via a skateboarding accident. Initial radiographs showed no evidence of acute fracture, and CT imaging was performed. CT scan of the right lower leg found a Tillaux fracture of the distal right tibia with a 2 mm displacement and a nondisplaced Salter-Harris type III distal fibula fracture. Closed reduction and percutaneous screw fixation of the distal tibia fracture were performed. The repair of this fracture was complicated due to the presence of two distinct fractures. This case study aims to provide a viable option to successfully repair this complex presentation as well as explain imaging findings that differentiate this fracture from other pathologies that are not managed operatively.

## Introduction

Tillaux fractures are infrequent adolescent fractures that are rarely covered in the clinical literature. These fractures are a type of Salter-Harris type III fracture that occurs most commonly from supination and external rotation of the ankle. External rotation of the foot relative to the leg causes tightening of the anterior talofibular ligament (ATFL) and can cause avulsion fractures of the tibial attachment. This mechanism causes a fracture through the anterolateral aspect of the distal tibial epiphysis and can represent 2.9% of juvenile epiphyseal growth plate injuries [1]. The maturation pattern of the physis contributes to these injuries as the central portion of the physeal plate closes first, followed by the anterior and medial portions, and finally, the anterolateral portion. When the ankle experiences extreme external rotation and supination, the weakest point, located along the anterolateral portion, fractures first. Because of the asymmetrical closure, the fracture continues through the open anterolateral portion of the physis until it reaches the closed segment medially. Once the fracture reaches the closed segment, it is then directed toward the epiphysis of the distal tibia, creating the characteristic type III Salter-Harris fracture. This mechanism unfolds through the ATFL, which causes avulsion as it pulls on the anterolateral corner of the distal tibial epiphysis, resulting in variable displacements. Current guidelines suggest that treatment is variable depending on the significance of the displacement. The standard protocol for a displacement less than 2 mm includes a nonoperative closed reduction to facilitate healing. Fractures with a displacement greater than 2 mm should be managed operatively [2].

The major signs and symptoms of this injury include pain and swelling in the anterolateral compartment of the ankle. This presents in a fashion similar to a common ankle sprain occurring through the ATFL. Tillaux fractures and ATFL sprains occur predominantly in athletes, as this population tends to experience increased stress on the ankle joint [3]. Adolescents presenting with these symptoms should undergo radiographic imaging for potential Tillaux fractures. Radiographic findings are typically remarkable for a vertical fracture through the distal tibial epiphysis with extension horizontally through the lateral aspect of the physis. Tillaux fractures are very similar to tri-planar fractures but can be distinguished by the lack of metaphyseal fracture present in the coronal plane [1]. The goal of this case presentation is to provide a repair option for a complex multifracture involving a Tillaux fracture. This study aims to validate the use of CT imaging in diagnosing Tillaux fractures and rule out other ankle injuries with complicated presentations.

## Case presentation

**Figure 1:**
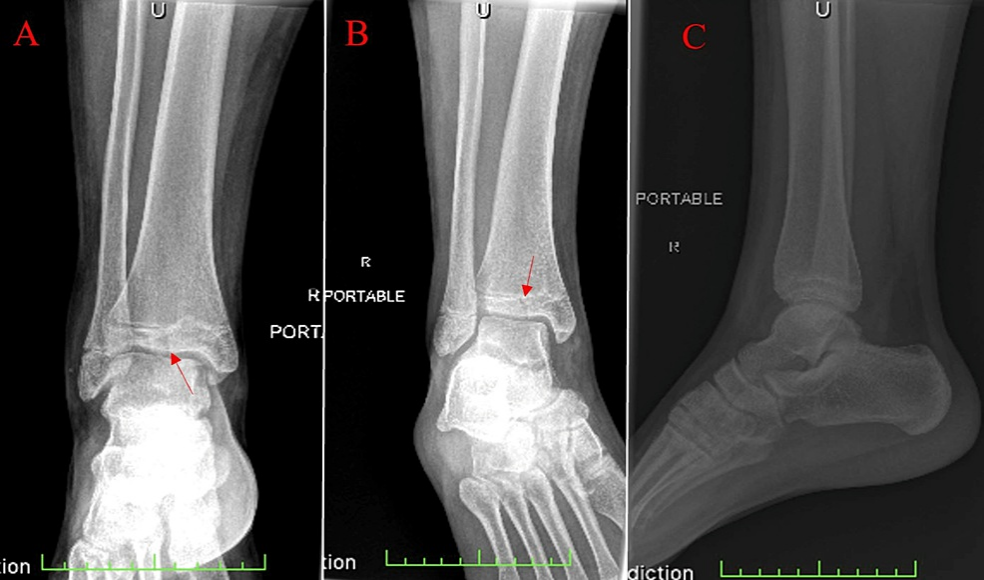
Plain radiographic images from the patient's initial visit to the emergency department. (A) AP view, (B) internal oblique view, and (C) lateral view. The red arrow indicates the single vertical lucency through the distal tibial epiphysis. Incomplete closure of the tibial physis can be appreciated. AP: anterior-posterior.

A 16-year-old male patient presented to the emergency department with the chief complaint of pain in the lower right ankle with prominent swelling. He stated that the pain began after falling off his skateboard, and swelling followed quickly thereafter. On physical examination, range of motion was significantly limited in eversion and inversion, and pain was elicited with light palpation. An initial three views of the right ankle were obtained via plain radiograph films. Findings demonstrated normal osseous density with an incomplete fusion of the physis. Ankle mortises were symmetric bilaterally, and a small joint effusion was present. A frontal radiographic view of the ankle demonstrated a vertical lucency through the distal tibial epiphysis (Figure 1). Initial impressions of the imaging were thought to represent no evidence of an acute fracture, and the single lucency through the distal epiphysis was representative of an artifact. CT imaging was ordered for better visualization as an outpatient.

**Figure 2:**
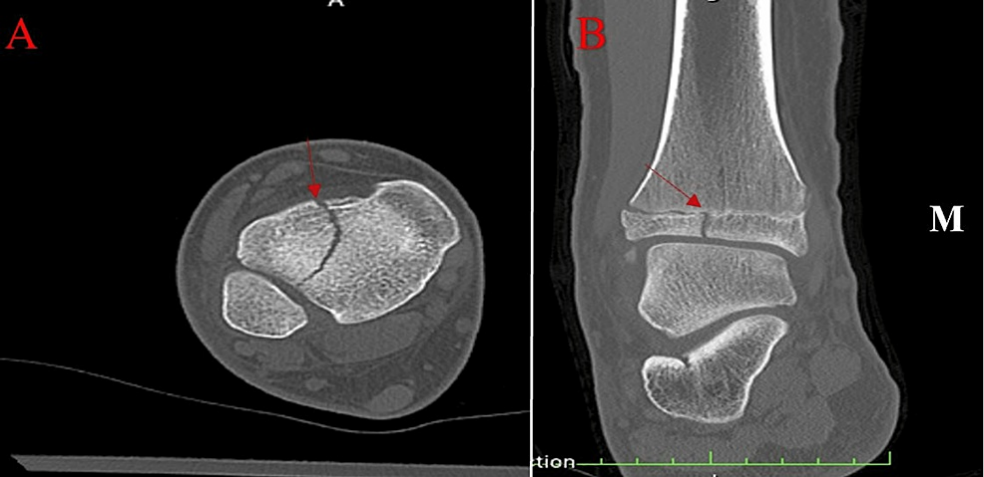
CT scan of the right lower extremity. (A) An axial view demonstrating the vertical fracture through the anterolateral tibia. (B) A coronal cut of the distal tibia. A red arrow highlights the fracture in both images. Displacement can be visualized on the lateral side, and this fracture pattern is indicative of a Tillaux fracture.

**Figure 3:**
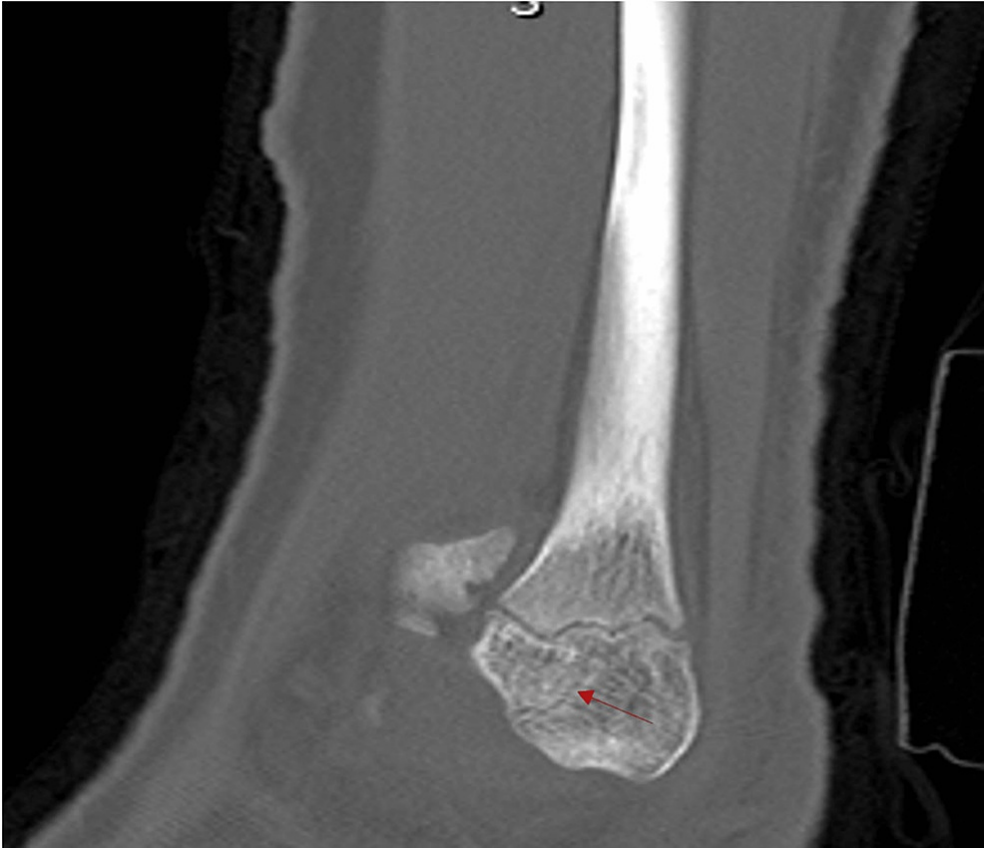
Sagittal view of a CT scan showing a nondisplaced Salter-Harris type III fracture of the fibula. A red arrow indicates the fibular fracture located at the anteroinferior margin of the epiphysis.

A lower extremity CT without contrast was conducted nine days after the initial injury. Unenhanced helical axial CT images with high-resolution orthogonal reconstructed images helped identify the patient’s source of pain. Radiation dose reduction techniques were used throughout this process, and the dose length product was calculated at 157.6 mGy/cm. The lucency noted on radiographs was identified as an acute fracture, and the break pattern was further classified as a Salter-Harris type III injury with a visible widening of the tibial physis that extended laterally (Figure 2). A second fracture line was isolated and found to be extending out of the anterior aspect of the tibia, including the distal-most 25% of the anterolateral tibial epiphysis. CT imaging also showed an irregularity of the fibular physis near its lateral margin. The sagittal images demonstrated a small trabecular lucency extending out the anteroinferior margin of the epiphysis (Figure 3). This finding was indicative of an additional Salter-Harris type III fracture of the fibula without displacement. The combination of a Tillaux fracture and a Salter-Harris fracture of the tibia is rare. To aid in surgical planning, a 3D image of the patient's lower extremity was digitally created.

The findings from the constructed 3D imaging showed a fragment displacement of just greater than 2 mm, indicating that closed reduction and percutaneous screw fixation were necessary. The patient was taken to the operating room two weeks after the CT findings for closed reduction percutaneous pinning (CRPP) of the fractures. During the operation, the Tillaux fracture was identified with C-arm fluoroscopy, and a linear bone clamp was placed across the distal tibia and closed, creating a reduction of the fracture. The patient's foot was placed in dorsiflexion followed by internal rotation to aid reduction of the fracture. Using C-arm fluoroscopy, a K-wire was placed across the tibia, just distal to the growth plate. The K-wire was used to approximate depth and size following the standard AO surgery technique. A 5.0 mm partially threaded cannulated screw was placed across the K-wire with a washer, resulting in excellent reduction and fixation of the distal tibia. The reduction and fixation were performed medially to laterally and were confirmed with C-arm fluoroscopy in the anterior-posterior (AP) and lateral planes. 

**Figure 4:**
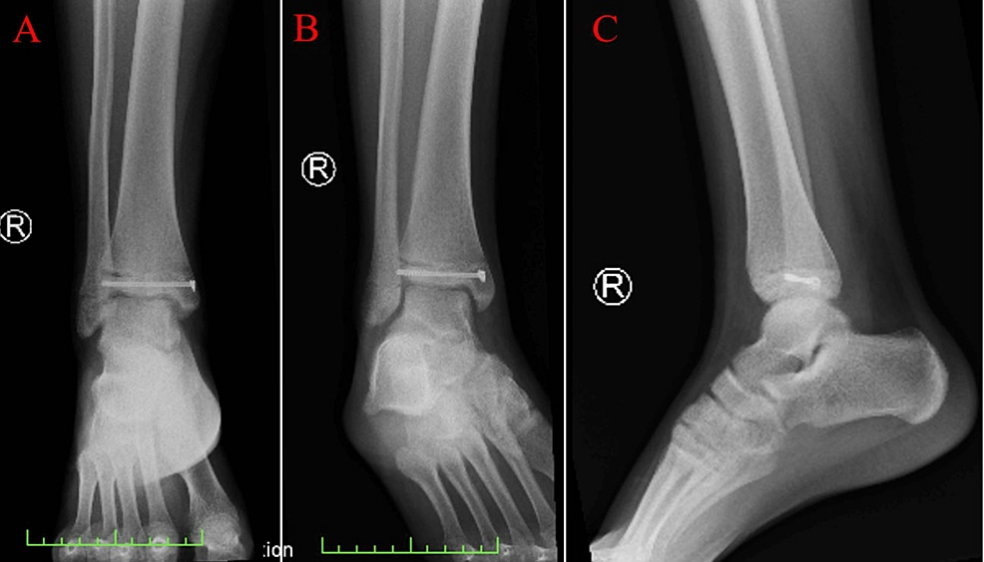
Plain radiographic images post-reduction and fixation of the fracture. (A) Postoperative AP view, (B) AP oblique with internal rotation, and (C) lateral view. The cannulated screw, reducing the displacement located within the tibia, can be appreciated in each image. AP: anterior-posterior.

Plain radiographic imaging captured post-surgical intervention shows the 5.0 mm partially threaded cannulated screw, and the appropriate reduction and fixation of the fracture were appreciated (Figure 4). The patient was placed in a long leg cast for four weeks to control the rotational component of the injury. Immobilization was also indicated for the healing of the Salter-Harris type III fracture in the fibula. The patient followed up in the outpatient clinic four weeks after the procedure to remove his cast. Both fractures were completely healed at this time. He reported no pain on palpitation or motion. The patient experienced post-surgical weakness, which was treated with physical therapy for four weeks, and he was able to regain his baseline strength in the lower leg and ankle. The patient made a full recovery but has been advised to watch for any complications, such as degenerative arthritis and varus deformity, which could potentially occur over time.

## Discussion

Tillaux fractures with ipsilateral, Salter-Harris type III fibular fractures are relatively uncommon injuries and have only been mentioned a few times in orthopedic literature, along with a variety of treatment options [4-6]. The strength of the ATFL and the open tibial physis during early adolescence creates an environment for Tillaux fractures, which occur exclusively in this age group [7]. High suspicion should be held for a Tillaux fracture in adolescents with swelling of the anterior aspect of the ankle with decreased ability to bear weight [2]. This presentation is synonymous with that of a lateral ankle sprain and can be missed clinically if the clinician is only relying on symptoms to diagnose. Improper diagnosis of a Tillaux fracture can lead to chronic complications, and appropriate diagnosis and treatment are essential. 

These fractures are difficult to diagnose due to the challenge they pose to see on imaging [4]. In this case, a CT scan was required to clearly define the lucency as a Tillaux fracture. The ability to identify a potential Tillaux fracture based on the initial presentation is of the utmost importance. These fractures are commonly missed and often misdiagnosed as mild ankle sprains [6]. CT scans are typically underutilized; however, they are especially indicated for ankle injuries in patients aged 12-16 years. Orthopedic surgeons should consider a CT scan when presented with a patient complaining of anterior compartment swelling of the ankle and benign findings on X-ray, so as not to misdiagnose or miss an indolent Tillaux fracture. X-rays are unable to identify the lucency accurately when compared to a CT. Going forward, CT should be the standard approach of imaging when an adolescent presents with lateral ankle injuries. Combined fractures, such as this, can be severe if missed and can cause long-term complications such as malunion, osteonecrosis of the distal tibial epiphysis, early onset arthritis, and premature growth arrest. The development of these complications would permanently alter the patient’s life. Future surgeries would be unlikely to correct the avoidable abnormalities created by the lack of diagnosis. 

Current literature emphasizes the use of ORIF to repair Tillaux fractures and fractures occurring along the medial malleolus [5,8]. The fracture pattern presented in this case further emphasizes the unique nature of the fracture, as this patient had a concurrent Salter-Harris type III fracture in the lateral malleolus, which was repaired with CRPP using a 5.0 mm partially cannulated screw. ORIF creates too many unnecessary complications, including a higher fixation failure rate and increased pain in patients following the procedure [9]. CRPP with a cannulated screw was able to correct the displacement effectively and efficiently without needing to open the skin. 

## Conclusions

A Tillaux fracture in an adolescent with a concomitant, nondisplaced Salter-Harris type III fracture in the distal fibula is a novel presentation of this rare fracture. Adolescents presenting with anterior swelling in the ankle should be examined for Tillaux fractures. One must consider the mechanism of injury, the patient’s age, and the location of the injury to best stratify the risk of this diagnosis. These injuries have been misdiagnosed as simple ankle sprains in previously reported case studies; thus, appropriate imaging should be conducted in high-risk patients for a clear diagnosis. Physicians should be cautious to exclude Tillaux fractures in adolescents if X-ray findings are inconclusive. CT studies should be utilized in this population in order to rule out potentially missed Tillaux fractures. To avoid potential complications and increased morbidity/mortality for the patient, as well as improve the patient’s quality of life, it is imperative that a proper diagnosis be made accurately and efficiently. Using the described surgical procedure, Tillaux fractures can be repaired while minimizing the surgical risks experienced in other procedural methods.
